# Scientific supremacy as an obstacle to establishing and sustaining interdisciplinary dialogue across knowledge paradigms in health care and medicine

**DOI:** 10.1007/s11019-019-09901-x

**Published:** 2019-04-25

**Authors:** Birgitta Haga Gripsrud, Kari Nyheim Solbrække

**Affiliations:** 1 Department of Caring and Ethics, Faculty of Health Sciences, University of Stavanger, Postboks 8600, Forus, 4036 Stavanger, Norway; 2 Professional Relationships in Welfare Professions Research Group, Department of Caring and Ethics, Faculty of Health Sciences, University of Stavanger, Stavanger, Norway; 3 Stavanger Breast Cancer Research Group, Stavanger University Hospital, Stavanger, Norway; 4 Department of Interdisciplinary Health Sciences, Faculty of Medicine, University of Oslo, Oslo, Norway; 5 Society, Health and Power (SHEP) Research Group, Department of Interdisciplinary Health Sciences, Faculty of Medicine, University of Oslo, Oslo, Norway

**Keywords:** Hereditary breast cancer, Interdisciplinarity, Scientific supremacy, Responsible research and innovation, Ethics, Different realities

## Abstract

This is a response to a short communication on our research presented in Solbrække et al. (Med Health Care Philos 20(1):89–103, 2017), which raises a series of serious allegations. Our article explored the rise of ‘the breast cancer gene’ as a field of medical, cultural and personal knowledge. We used the concept *biological citizenship* to elucidate representations of, and experiences with, hereditary breast cancer in a Norwegian context, addressing a research deficit. In our response to Møller and Hovig’s (Med Health Care Philos 21(2):239–242, 2018a) opinionated piece, we start by questioning on which scientific grounds they base their knowledge claims and situate their criticism in a pre-determined positivist script, which exposes their incompetency when it comes to establishing a useful critique of our research. We tie this to an attitude of scientific supremacy, which reduces the complexity and specificity of different knowledges into a clichéd divide between ‘hard evidence’ and ‘fiction’—presented in a predictable narrative which seeks to establish research protagonists and antagonists. We elaborate on the rationale of our qualitative approach to analyzing and interpreting situated and mediated aspects of BRCA 1/2. We counter claims that our research does harm to patients. We refer to a medical scandal emerging from Norway where 21 women were wrongfully diagnosed and surgically treated for a mis-interpreted cancer gene mutation. In conclusion, we stand by the integrity of our research as reported in the original paper. Scientific supremacy and pre-scripted criticism impose considerable obstacles for the possibility of establishing interdisciplinary dialogue across knowledge paradigms in health care and medicine. We therefore urge readers to reflect on how we can establish and sustain ethically careful and truthful dialogue—without doing violence to epistemological differences—to protect and advance the interdisciplinarity that constitutes the journal’s scope.

## Background and introduction

This is a response to Møller and Hovig’s (2018a) short communication on our original research article in *Medicine, Health Care and Philosophy* (Solbrække et al. 2017). In our 2017 article we explored the rise of what has popularly been called ‘the breast cancer gene’—as a field of medical, cultural and personal knowledge. We used the theoretical concept *biological citizenship* (Rose 2007) to elucidate representations of, and lived experiences with, gene testing technology and so-called ‘hereditary breast cancer’ (Møller et al. 2005) in a Norwegian public healthcare context, addressing a national research deficit. Møller and Hovig’s commentary contains numerous serious allegations, mis-representations and mis-interpretations of our research, the most grave of which we would like to address and correct on behalf of our interdisciplinary author group through this response.

Møller and Hovig claim that our 2017 article relates to a ‘reality […] so different from ours that it is tempting to cite the current sociopolitical notion of ‘alternative facts’’ (pp. 239–240). We concur that it does indeed appear as if we are relating to two different perceptions of reality. However, we reject the insinuation that our study represents ‘alternative facts’. One obvious explanation of our reality discrepancy is that we inhabit quite different knowledge-positions within health care and medicine, however such a difference is not acknowledged by Møller and Hovig, who do not appear capable of any outlook beyond their own epistemological position and scientific interest. Thus, the premise for establishing a constructive interdisciplinary dialogue and criticism is severely compromised from the outset. Not that Møller and Hovig’s commentary give us any reason to think that they invite such dialogue. In terms of its form, Møller and Hovig’s short communication is presented in what can only be characterized as a verbally excessive and haphazardly manner. A balanced tone is only achieved when it comes to presenting their own research interests and stakes in the topic. In terms of its content, the short communication presents a series of serious claims against our study. For example, Møller and Hovig (2018a) conclude that to them our paper ‘is not scientifically valid’ (p. 242), stating that what we present in the study are ‘alternative facts’ (p. 239), ‘what you see is what you look for’ (p. 239), and ‘fiction’ (p. 240), as well as ‘opportunistic highly selected arguments for a group constructed on sociopolitical, sexual and emotional reasons […] commonly referred to as identity politics’ (p. 242). Further to this, they claim that our article not only challenges Human Rights, but could be ‘disease-creating, by telling the patients how dreadfully difficult their lives are’ (p. 241), thereby violating ‘the philosophical rule of not doing harm’ to patients (p. 239). In response, we wish to ask: on which scientific grounds do Møller and Hovig base these claims? We question their competency, as geneticists, to assess the scientific integrity and value of our qualitative inquiry. In fact, it is hard to see how *any* qualitative research could *ever* be adequately evaluated, according to Møller and Hovig’s *implicit* quality criteria, which appear to be firmly based in the positivist empirical tradition belonging to the natural sciences. As regards most of their allegations (‘not scientifically valid’, ‘alternative facts’, ‘what you see is what you look for’ and our research as ‘fiction’), we can only reject them because they are ill-founded and incorrect. In what follows we shall clarify why this is so.

But let us start by clarifying what kind of research *we* do, in the spirit of encouraging shared understanding and mutual respect for epistemological differences. Qualitative research entails the collection and study of various empirical materials: personal experience, life-stories, case studies, artifacts, visual texts, cultural texts and events, as well as historical and observational texts (see Denzin and Lincoln 2018). These empirical materials concern meanings and human meaning-making. Because qualitative research is concerned with meanings, it is largely an *interpretative practice*. As qualitative researchers we have both engaged with detailed analysis and interpretation of the data the article draws on, and these cannot be said to represent ‘opportunistic highly selected arguments’, as Møller and Hovig claim (2018a, p. 242). As Møller and Hovig appear to have spotted, identity is a significant area of interest within qualitative research because it concerns the complex psychosocial interface between personal experience and subjectivity—and the social and cultural environment in which we live our lives. Identity concerns who we become in relations with others, and through finding our place in society. However, an interest in identity as an object of qualitative inquiry cannot be conflated with ‘identity politics’ (Møller and Hovig 2018a, p. 242). We can assure Møller and Hovig that as researchers interested in representations and narratives of hereditary cancer, we do not represent any agenda on behalf of a group ‘constructed on sociopolitical, sexual and emotional reasons’ (ibid.), other than that we are women and as such belong to a group that makes up a little under half of the world’s population.

Qualitative research has a long history and has become an established branch of human and social sciences. Resistance to qualitative research is not new. It is recognizable in the long-standing debates concerning clichéd divides between “hard” and “soft” sciences, which divides researchers into roles as protagonists or antagonists of scientific progress. In light of such longstanding debates, it is easy to spot how Møller and Hovig’s commentary adheres to a predictable standard script. For example, Denzin and Lincoln (2018, p. 8) refer to the recurrent allegations that qualitative researchers are ‘soft scientists’, whose work is ‘unscientific […] or subjective’. In this pre-scripted plot experimental positivist sciences are ‘seen as the crowning achievements of Western civilization’ (ibid.), and anything outside it must therefore be a disappointment. In positivist scientific discourse, ‘it is assumed that “truth” can transcend opinion and personal bias’ (ibid.), resulting in the idea of a ‘value-free objectivist science’. In contrast to this, qualitative research is seen by positivist critics ‘as an assault’ on scientific tradition (ibid.). A common allegation by researchers within the positivist scientific paradigm is therefore that ‘qualitative researchers write *fiction*, not science’ (ibid., our emphasis). We will return to this issue, when we discuss the detrimental consequences of scientific supremacy, which reduces the complexity and specificity of knowledge into a clichéd divide between “hard evidence” and “alternative facts”. By inserting their commentary on our article into this pre-designed but worn-out script, Møller and Hovig expose their incompetency when it comes to understanding the fundamental aspects of qualitative research approaches and the differences between our epistemological paradigms. Hence, as we see it, they cannot lay claim to a position of scientific authority when it comes to assessing the quality of our research. What remains to be responded to then, cannot be considered as a scientifically valid criticism of our research. Rather our response must concern addressing Møller and Hovig’s *opinions* about our research, which they are fully entitled to. In what follows we will address the scientific attitude evoked by Møller and Hovig’s short communication, before moving on to the rationale for our chosen mode of inquiry into BRCA 1/2. We respond to Møller and Hovig’s specific criticism that our study does not consider the different ethos of US versus Norwegian healthcare regimes. Further to this, we respond to Møller and Hovig’s allegations that our research challenges universal Human Rights, ‘is disease-creating’ and ‘does harm’ to patients—by reference to a recent case of medical malpractice in Norway. We conclude on a note about the need for reflection about the ethics at stake in dialogues across different scientific knowledge positions, productions and practices—particularly in an *interdisciplinary* journal for health care, medicine and philosophy.

## An attitude of scientific supremacy versus intersecting and entangled knowledges

We have noted above how Møller and Hovig’s commentary reveals limited awareness of the qualitative paradigm that frames our study. The sociologist Pierre Bourdieu once noted how formidable barriers lie ‘in the fact that the solidarity that binds scientists to their science (and to the social privilege which makes it possible and which it justifies or procures) predisposes them to profess the superiority of their knowledge’ (1990, p. 28). In our view, such an attitude of scientific supremacy, deeply anchored in the bind to a particularly privileged discipline such as biomedicine, is a characteristic feature of Møller and Hovig’s short communication. The supremacy attitude is revealed by a fundamental lack of respect for a different knowledge-position and a lack of curiosity or will to engage in a constructive dialogue across such divergent knowledge-positions. For example, they totally overlook the fact that when we introduce our topic and discuss our findings, we are on a theoretically-based ground that extends beyond a dichotomous positive/negative view on the rise of genetic testing. We argue *unambiguously* that ‘there is little doubt that medical innovations, including genetic testing, have broadly contributed to significant health improvements’ (Solbrække et al. 2017, p. 99). However, epidemiology and public health were not our concern in this paper. Rather, we wanted to pursue Rose’s (2001) prophetic assertion that ‘gene testing is not just an unavoidable aspect of modern society but also *the knowledge that makes us what we are*—that is, technoscience and medicine are what provide us with our bio-identities’ (quoted in Solbrække et al. 2017, p. 99). We did so by highlighting ‘the ways in which knowledge about breast cancer gene mutations shape the subjectivities of the individuals who are left to deal with the potential consequences, practically, aesthetically, emotionally, physically, existentially and relationally’ (ibid.) Møller and Hovig’s verdict is simply that we ‘have misunderstood the concept of biological citizenship’ (2018a, p. 242). We therefore wish to clarify any misunderstanding, whether ours or theirs, by elaborating on the rationale of our approach to studying genetically inherited cancers. We do so to clarify our epistemological knowledge position and illustrate how it differs in substantial ways from that of the positivist empiricism traditionally associated with biomedical science.

Knowledge about genetic dispositions for disease is a prime example of how life sciences, biotechnology and biomedicine intersect. According to the sociologist Nikolas Rose (2001), whose theories we draw on in our 2017 article, this intersection has affected the understanding of what it means to be a human being, through a somatic turn where identity is increasingly defined as corporeality, making new links between biology and behavior. This is the analysis which leads Rose to coin the term ‘biological citizenship’. On the basis of Rose’s theoretical work, in our article, we were curious to explore how being a carrier of a gene mutation might come to shape human subjectivity—within one national context. Our curiosity emerged from discussions in the author group about what we assumed to be a globalised American discourse on BRCA 1/2 and so-called breast cancer previvorship, exemplified by media representations of the US celebrity Angelina Jolie, and speculations about an ‘Angelina Jolie effect’ (see e.g. Theissen 2015; Thue 2015) across the western hemisphere, leading many more women to seek out diagnostic genetic testing for BRCA 1/2. The speculation about the Angeline Jolie effect on women’s attitudes to genetic testing has now been confirmed in a review study that reports an increase of referrals to BRCA-testing, with a peak of +80 percent (Troiano et al. 2017). Exploring and discussing the possible connections and contrasts between a globalised previvor-discourse, and representations and experiences emerging from a national context became one of the premises of our study. Perhaps we failed to communicate this clearly enough, and this is the reason why Møller and Hovig (2018a) claim that we do not understand how US (private) health care and Norwegian (public) welfare practices differ in their ethos. We would like to respond to this claim by saying that we were not unaware of these health care discrepancies but that differences between health and welfare systems were not the object of our inquiry. We were concerned with whether globalised discourses on BRCA (e.g. Jolie’s outspoken previvor empowerment narrative) materialised in national media representations and narratives in a Norwegian context. We found this not to be the case. Hence our study contributes new knowledge about what may be a particular form of “Norwegian previvorship” (and *survivorship* for the three interview participants who ended up developing a genetically inherited breast cancer).

As mentioned in Solbrække et al. (2017), the outline of our article was initially laid down during a research seminar that brought together Norwegian researchers to discuss critical approaches to cancer survivorship. This collaboration has since evolved into a large Research Council of Norway financed project entitled Rethinking Cancer Survivorship (Project No.: 283517), an indication that the scientific and societal value of such critical approaches is recognized by the peer community. The methodological approach behind our 2017 study is aligned with the cultural studies tradition for interpretative bricolage: “a pieced-together set of representations that are fitted to the specifics of a complex situation” (Denzin and Lincoln 2018, p. 11). For our exploration we used interview data (n = 3) from a larger qualitative study (n = 14) on women’s experiences of breast cancer in Norway, in which three participants turned out to have an underlying BRCA-mutation. We also referred to the global media attention surrounding Angelina Jolie’s openness about her BRCA1-diagnosis and prophylactic surgeries, as well as a newspaper interview with a young Norwegian woman carrying a BRCA-mutation and who had opted for full prophylactic surgery. Due to the interdisciplinary composition of our author group, which included a medical professor and senior breast cancer consultant, we also referred to biomedical literature in our background chapter on hereditary breast cancers, paying due respect to Pål Møller’s contributions to the field. In other words, in acknowledgment of the bio-psycho-socio-cultural complexity of inherited cancer, we strived to establish an accessible but medically accurate presentation of BRCA 1/2 before launching into our investigation.

One of our main findings is that, despite its claims as a measure for health, knowledge of ‘the breast cancer gene’ can also produce severe instability in those affected by it (Solbrække et al. 2017). This was particularly evident in the newspaper interview we refer to where Louise Skak told the reporter how she was constantly on the alert, surveilling her body for signs of any malignancies. Despite having had a prophylactic oophorectomy, mastectomy *and* hysterectomy, she did not appear reassured of her health (Solbrække et al. 2017, p. 94). Although gene testing provides modern subjects with an opportunity to foresee their biological destiny and undertake pre-emptive measures to prevent severe illness and premature death, it may also introduce existential dilemmas that resonate beyond the individual and into different relationships. To illustrate this, in one of our article’s participant narratives, the woman we call ‘Bente’ expresses a strong feeling of guilt that she, as a mother, has introduced the problems of gene mutations, breast cancer and genetic testing into her teenage daughter’s life at a stage when she is just about to enter womanhood. We interpreted this as an indication of how having genetic knowledge can be experienced not only as safety from harm but also as an existential point of no return, as well as a considerable relational burden. Hence, our allusion to ‘a loss of innocence’ (Solbrække et al. 2017, p. 98) and eating the fruit from the tree of knowledge, to which Møller and Hovig give disproportional consideration in their commentary (see 2018a, p. 241). As all three interview participants reflected on similar dilemmas, it led us to coin the concept *families in waiting* (a spin-off from Timmermans and Berg’s (2010) ‘patients-in-waiting’) as an apt description of gene testing’s complex relational, generational and emotional transmissions and tensions. When we engaged with the data, we did so, not from the perspective of clinical medicine, nor with the aim to generalize from our small sample and eclectic data. Rather we engaged with the material at hand using our knowledge and pre-understanding as qualitative researchers concerned with illness narratives (see e.g. Charon 2006; Frank 1995). Our previous research has, for example, studied how people with a diagnosis or illness try to make sense of their experiences (see e.g., Gripsrud et al. 2016, 2014; Solbrække and Bondevik 2015). This kind of experiential and embodied meaning-making can be psychosocially complex (see e.g., Gripsrud et al. 2018), rarely adhering to the Enlightenment ontology of pure rationality and a unitary thinking subject, nor to the mind/body split frequently associated with biomedicine. On the contrary, experiential meaning-making in the face of serious illness tends to be enmeshed, complicated and does not always stand up to reason—a truth that physicians face every day in clinical encounters with patients. Genetic consultations, specifically, may involve a particularly high degree of intricacy and complexity, because they entail

intense bi-directional affective entanglements between all the parties to the encounter, and indeed generate multiple ‘virtual’ entanglements with parties not present—distant relatives, absent siblings, potential offspring. In these entanglements, the ethical relations of all the subjects to themselves and to one another are at stake—including the experts themselves. The consultation acts as an intensifier of ethicality. It mobilizes affects of shame and guilt, and of the respective claims, scope and limits of freedoms for the self and obligations to others. It activates the conflicts within the counsellors between the ethics of care and the ethics of guidance. (Rose 2001, p. 10).

Only with awareness of this intricate entanglement of affects and relationships, caring and guidance, can one truly engage with the many existential and ethical layers produced by our topic and findings. The Rose quotation provides a sophisticated understanding of the difficult ethical layering involved in genetic consultations and testing, implicating not only the patient, but the patient’s relatives, as well as the experts and counsellors who must be careful in their clinical guidance. In this sense, Rose’s analysis defies a simplistic dichotomous set-up between patient autonomy and choice of treatment *or* medical paternalism (see Møller and Hovig 2018a, p. 240). By going into a detailed analysis and interpretation of our small but carefully selected data material, we could bring such ‘affective entanglements’ to light. According to Møller and Hovig our interest is ‘a few selected patients’ feelings’ studied outside of the ‘contextual settings’ (2018a, p. 241). To clarify our agenda, we were indeed concerned with contextual settings, but not with the particular setting imposed on us post hoc by Møller and Hovig. Our contextual setting was the way in which medicine branches out to, and is intertwined with, specific cultural practices, social dynamics and meaning systems, an understanding that has become widely accepted for the past thirty years (e.g. Green and Britten 1998; Kirkengen et al. 2016; Kristeva et al. 2018; Kleinmann 1988; Plsek and Greenhalgh 2001; Greenhalgh et al. 2016). It has been 15 years since Klawiter (2004) used the concept ‘disease regimes’ to describe the intricate interplay between social structures, cultural factors and subjective life experiences related to breast cancer. In our study we similarly used a critical-interpretative bricolage approach to identify ‘disease regimes’ related to both prevention, and curative treatment of, genetically inherited breast cancer. We did this, not in order ‘to point out certain thought trails as more true or better than others, but to view and explain the logic of different and conflicting thought trails’ (Fossåskaret and Aase 2014, p. 61, our translation from Norwegian). This is the very epistemological proposition of most qualitative research. As we developed our analyses of conflicting trails in our data, we did so with a degree of humility, based on the understanding that knowledge, whether emerging from narratives or numbers, is never final and complete, but rather must always be considered as multi-stranded, situated and preliminary. The result was a paper which, unbeknownst to Møller and Hovig, was ‘accepted as is’ by the journal’s two academic referees, which we consider as a solid peer community acknowledgement of its scientific value.

## The ‘philosophical rule of not doing harm’

In their commentary Møller and Hovig (2018a), claim that ‘patients feel harmed’ by our position and that we are ‘violating the philosophical rule of not doing harm’ to patients (p. 241). These are claims that we reject, firstly because Møller and Hovig have no empirical basis to say that ‘patients feel harmed’ by our article. If this is so, we would like to talk to them. Secondly, as we see it, one cannot do harm to patients simply by providing a nuanced account of mediated representations and narratives of lived experience. On the contrary, we would argue, it is for the better of patients and clinicians, that the scientific and medical communities understand more about the experiential complexities and challenges facing this patient group. Norwegian legislation stipulates that a patient’s human complexities must be of concern for health care staff who, according to §4 of the ‘Act relating to health personnel’, are under the legal obligation to provide ‘sound professional and considerate care’ (Norwegian Ministry of Health and Care Services 1997; Braut 2011). In this context, our research may contribute informing such sound and considerate care, thereby supporting the ethical medical principles of beneficence rather than jeopardizing patient safety.

As far as doing harm to patients is concerned, we would claim that the stakes can be much higher in biomedical science and medical practice than in qualitative inquiry into illness experience, as will become evident when we look at a recent case from Norway. In 2017, a newspaper exposé revealed how at least 21 patients at Oslo University Hospital had been subject to medical maltreatment in the period between 2002 and 2014 (Storvik 2017a). The women had been told by genetic advisors that they had a cancer risk related to a gene mutation, and that a recommended course of action would be to remove their ovaries and/or breasts. But it turned out that their genetic tests had been misinterpreted: their results were now seen as false positives. The mistake was discovered in 2014 but not reported to the health authorities as a serious incident until 2 years had passed (Storvik 2017b). The 21 women were subsequently informed by the hospital that there had been no valid scientific basis for their prophylactic surgeries. Whistleblowing from the genetics department at Bergen University Hospital eventually led to a health authority audit of Oslo University Hospital (Olsen 2017b). While this was going on in 2017, Pål Møller and Eivind Hovig decided to publish a scientific article *defending* the previous interpretation of this particular gene mutation as a cancer risk factor. However, this article was subsequently retracted in May 2018, ‘due to coding errors’ (Møller and Hovig 2018b). The article was heavily criticized by genetic researchers at Bergen University Hospital (Stranden 2018a), illustrating the depth of a longstanding dispute between different research communities in Norway, that we referred to in our article (Solbrække et al. 2017) to the apparent insult of Møller and Hovig (2018a). In 2018, the health authority audit concluded that Oslo University Hospital had acted medically irresponsible towards the patients in question (Stranden 2018b). In the light of this case, Møller and Hovig’s opinions of our study appear even more disproportionate. Their accusatory commentary was first published open access online, ahead of print in the autumn of 2017, under the identical heading of our own article (an effective coup). Their insinuations that *our* study does harm to patients and is ‘disease-creating’ (Møller and Hovig 2018a, p. 241) now appear somewhat absurd, as Møller and Hovig and the scientific community to which they belong have become thoroughly embroiled in scientific and medical controversies with very high stakes for the unfortunate patients at the receiving end. Møller and Hovig write in their commentary that the ‘only interesting problem we find in Solbrække et al.’s paper is their interest in female breasts, and which is an interest they have not explained’ (2018a, p. 241). If Møller and Hovig are still struggling to understand why we focus on what it entails emotionally, reproductively, sexually and relationally for women to lose their breasts (and ovaries), we recommend that they read the newspaper interviews with some of the 21 women who had theirs’ removed unnecessarily due to unsound treatment at Oslo University Hospital (Olsen 2017a).

## Concluding remarks

What can we learn from Møller and Hovig’s short communication, other than that we are worlds apart? The main lesson is that scientific supremacy, of which they appear as staunch proponents, exacerbates the need to discuss the scientific and biomedical practices and ethics of care surrounding genetic testing in the Norwegian public health services. Our case reveals how imperative it is that societal and ethical aspects of genetic knowledge are brought to the fore in research. One strategy to fulfill this is the principle of Responsible Research and Innovation (RRI), which addresses the need for science that engages with civil society’s expectations, values and needs (European Commission 2018). We believe our own study from 2017 to be an example of how a biomedical topic can broadened out in this way. As researchers working at the intersection of disciplines, we notice increased acknowledgement of the fact that implementation of medical technologies and treatments resonate outside their immediate contexts in the clinic and the lab. However, despite strong rationales and ideals for RRI and interdisciplinary health research—it may be easier said than done. The tone of Møller and Hovig’s response, provoked by our exploratory attempt to transcend traditional boundaries between social science, humanities and biomedicine, illustrate some of the obstacles which may confound future efforts to broaden the contextual understanding of illness and health. Having responded to Møller and Hovig’s opinions about our article (Solbrække et al. 2017), we now have an even stronger conviction that we are thoroughly justified in challenging conventional ideas about medical knowledge as something exclusively defined, produced and curated by biomedical scientists, a discourse from which an attitude of medical scientific supremacy springs. We will therefore continue to engage dialogues across different knowledge paradigms in health and medicine—an agenda to which our 2017 article adhered. Møller and Hovig make several severe allegations against the scientific value of our article. We conclude our response to these allegations by stating that we stand by the scientific integrity of our research procedures and findings as they were reported in our original paper—an integrity that was unambiguously acknowledged by the journal’s peer reviews and editorship. Further to this, we wish to end our response on a more constructive note—in an appeal to this journal’s readers. Our appeal concerns the need for thinking about conditions for more fruitful discussions of complexities and uncertainties across different knowledge paradigms and multiple realities, in line with this journal’s scope to promote interdisciplinarity in philosophy, health care and medicine. The question that remains to be thought about is how we can establish and sustain ethical dialogues of truth *and* caring (*veritas in caritate*)—without doing violence to our epistemological differences.
